# Tempters and Gluten-Free Diet

**DOI:** 10.3390/nu8120786

**Published:** 2016-12-03

**Authors:** Carlo Catassi, Alessio Fasano

**Affiliations:** 1 Department of Pediatrics, Università Politecnica delle Marche, Ancona 60121, Italy; 2 Pediatric Gastroenterology and Nutrition, MassGeneral Hospital for Children, Boston, MA 02129, USA

To the tempter that came to Him and said, “*If You are the Son of God, tell these stones to become bread*”. Jesus replied “*It is written: man shall not live on bread alone* …” (Matthew 4:4). Nowadays, many people, particularly in the Western world, have gone well beyond that holy recommendation. In a 2014 US Consumer Reports survey, 63% of participants said they felt that avoiding bread and other gluten-containing food would improve their physical or mental health. Moreover, in a 2015 Gallup Poll, 21% of Americans reported they tried to include gluten-free foods in their diet. The “fall” of gluten might be related to the increased awareness of gluten-related disorders and the recent surge of prevalence of celiac disease (CD) and non-celiac gluten sensitivity (NCGS). This trend could still be on the rise, particularly in areas of the world undergoing a progressive “Westernization” of the diet, as suggested here by Peña and coworkers who note that the diet of people living in Central America is shifting from a maize- to a wheat-based diet [1]. Not surprisingly in Mexico Ontiveros et al. report a 3.7% of people purposely adhering to a gluten-free diet (GFD). Many of these individuals could be affected by NCGS [2]; however, the frequency of this condition is difficult to ascertain due to lack of a biomarker. This is the reason why a group of experts of gluten-related disorders met in Salerno, Italy, to standardize the NCGS diagnostic criteria [3].

Despite the previously mentioned diagnostic uncertainties, well-designed studies suggest that NCGS is not only common but also more clinically variable than originally reported. In this *Special Issue*, three original articles add important pieces of information: (a) many patients diagnosed as having irritable bowel syndrome are clearly gluten-sensitive, and their symptoms could be adequately controlled with a gluten-free diet only [4]; (b) “skin NCGS” is described for the first time by Bonciolini and coworkers, in non-CD subjects with skin lesions similar both to eczema and psoriasis showing itching, presence of C3 at the dermoepidermal junction, and a rapid resolution of lesions when adopting the GFD [5]; (c) the description of “gluten psychosis” in a pre-pubertal child presenting with a severe hallucinatory manifestation that was clearly related to the ingestion of gluten-containing food, and showing complete resolution of symptoms after starting treatment with the GFD [6].

Why and how gluten proteins can trigger an abnormal response ending up with clinical symptoms, particularly at the brain level, is still poorly understood, but it is likely that mechanism/s other than the adaptive, HLA-related immune response observed in CD patients, may be involved. There is currently a special interest on the effect of gluten on the so-called gut-brain axis with two not mutually exclusive theories, both starting with an increased absorption of undigested gluten peptides. Once these peptides reach the lamina propria, they may get into circulation, cross the blood-brain barrier, and ultimately interact with opioid brain receptor so affecting the individual’s behavior, attention and social interaction. Alternatively, these gluten peptides may cause activation of immune cells that leaves the gut mucosa, and migrate to the brain where cause neuroinflammation leading to behavioral changes. Although these sequences of events remain to be proven, new pieces of the puzzle are becoming available. In this *Special Issue* Hollon and coworkers show that gliadin exposure induces an increase in intestinal permeability in all individuals, regardless of whether or not they have CD. The results of this study suggest that gluten exposure leads to altered barrier function in both CD and NCGS, resulting in an exaggerated increase in intestinal permeability [7]. 

The quoted references are just a sample of the high-quality papers published here. As Editors of now three Special Issues on Gluten-Related Disorders, in the years 2013, 2015 and 2016 respectively, we are grateful to the *Nutrients* Editors in Chief for giving so much visibility to our scientific community and to the *Nutrients* Editorial Staff for their professionalism in handling the manuscripts. Now the question is: would the “modern tempter” ask Jesus to transform some of the stones in gluten-free instead of regular bread? This is just a matter of speculation. From our modest point of view we can only applaud this increasing interest in gluten-related disorders, i.e., an array of conditions affecting a growing proportion of the general population. 
